# Unequal Occupational Mobilities Between Rural Migrant and Urban Resident Workers in Urban China

**DOI:** 10.3389/fsoc.2020.00055

**Published:** 2020-09-04

**Authors:** Chong Zhang

**Affiliations:** Department of Sociology, Durham University, Durham, United Kingdom

**Keywords:** social mobility, occupational mobility, inequality, *hukou* system, China, rural migrant

## Abstract

The current scholarship on inequality of occupational attainment between rural migrant workers (RMW) and urban resident workers (URW) is largely dominated by evidence suggesting a landscape of occupational segregation, whilst there is a lack of studies researching the equality of occupational mobility. To fill this gap, this study compares the occupational mobilities between RMW and URW in China's urban labor market. Three heatmaps are used to visualize the differences between these two groups in the outflow distributions of occupational mobility. The results show a marked disadvantage of RMW's mobility into white-collar occupations and a relatively high tendency for them to move to or to stay in the manual and agricultural occupations.

## Introduction

Inequality between rural migrant workers (RMW) and urban resident workers (URW) is a well-known significant issue in urban China. RMW are defined as those who still have an “agricultural” *hukou* status but work as non-agricultural workers in the urban areas, while URW are those who have non-agricultural *hukou* status in cities (China Labor Bulletin, 2020). China's *hukou* (household registration) system divides nationals into two types of populations who are legally required to register as either agricultural or non-agricultural. For the sake of rapid industrialization during the planned economy period, China's *hukou* system prioritized economic and social development in the urban areas, which led to a marked issue of rural-urban divide in China (Chan, 2009). Since free migration between rural and urban areas was permitted during the era of economic reform, a massive shift in rural-to-urban population migration occurred due to better job opportunities and improved economic life chances in urban areas. There has been a known phenomenon of massive rural-to-urban migration for job opportunities and economic life chances improvement. However, most of the rural migrants failed to change their *hukou* status to non-agricultural. Strictly speaking, those with an agricultural *hukou* status in cities are still classified as “temporary” or *de facto* residents in cities (Chan, 1996). With no entitlement to the state-supplied urban welfare benefits, these residents' lives will have been more difficult than those of urban residents and, consequently, their economic security and well-being would have been even more reliant on working (Chan, 1996, 1999). Additionally, rich empirical evidence (e.g., Chan, 1996, 2009; Li, 1999, 2004; Démurger et al., 2009; Liu, 2017) has consistently documented a pattern of occupational segregation in urban China, noting that RMW are more likely to take low-income and unskilled “3-D” (dangerous, dirty, and demeaning) manual labor jobs, whereas urban populations' occupations are generally highly skilled and exhibit good economic remuneration.

Whilst a general picture of unequal occupational attainment is known, the area of equality of occupational mobility between RMW and URW is still under-researched. Inequality of mobility for those who started from similar occupational levels could reveal RMW's risks of employment and economic insecurity (if RMW had a higher downward mobility rate) and their disadvantage of further economic life chances enhancement (if RMW had a lower chance to achieve upward mobility). There is some research comparing RMW's and URW's job-changing frequencies, however these studies can only give limited information on inequality of mobility. For example, using International Socio-Economic Index of Occupational Status (ISEI), Li (1999, 2004) studies firstly documented that RMW's first- and second-time job changes after their labor market entry showed significantly less ISEI increase than URW's. Li (1999, 2004) argues that this was mainly due to URW's better job security than RMW. Thus, URW only changed their jobs when they were offered a better position (i.e., upward mobility) (Ibid.). Fu and Tang (2016) case study on sanitation workers in Shenyang also shows that rural migrant sanitation workers changed their jobs much more frequently than the urban resident workers. Additionally, other studies (Li, 2010; Zhang, 2011) have also documented that RMW's relative job insecurity exists in both the state and non-state sectors. However, these studies on “job changing” could only give a vague indication of the possible inequality in occupational mobility. Changing jobs more frequently does not necessarily mean being disadvantaged. Although Li (1999, 2004) studies have used the ISEI to indicate whether the changes were upward, horizontal, or downward, his studies compared individuals' first and second job changes in their career lives rather than the mobility within a fixed period as those comparative social mobility studies do. It could be the case that workers managed to achieve upward mobility in a very short period of time but had gone through many job changes in order to achieve this. To indicate the extent of equality of occupational mobility, a study ideally needs to compare RMW's and URW's occupational mobilities within a fixed period.

In addition, there are some studies researching the *hukou* effect on social mobility in general following a more rigorous comparative social mobility analysis approach, although those works are not confined to the context of urban China. Without controlling for workers' original occupations, both Wang (2003) and Li and Zhao (2017) found that agricultural populations had a lower risk of downward mobility, since most agricultural populations' occupations were already in the lowest level stratum (i.e., agricultural workers) in the used class scheme. However, they have different findings on upward mobility. Whilst Wang (2003) shows that agricultural populations had a lower upward mobility rate, Li and Zhao (2017) found the opposite. Without controlling for individuals' original social classes, these two studies are not particularly useful in highlighting the situation of equality in mobility. Furthermore, Wu and Treiman (2007) show that, compared to non-agricultural populations, agricultural populations were very likely to stay in or downgrade to agricultural occupations, unless they had previously achieved a good level of educational attainment. Likewise, Lu (2008) also documented that *hukou* status affects one's prospects of upward career progression. These two studies provide a more rigid comparison on social mobility between the overall agricultural and non-agricultural populations, and still provide very little knowledge about the difference between RMW and URW in the urban labor market. Even though rural migrants are regarded as socio-economically disadvantaged populations in urban areas, they are actually the more advantaged among those who hold an agricultural *hukou* status (Li, 2000, p. 127–129; Li, 2004, p. 34). In other words, RMW are not a random sub-group of agricultural populations but a complex grouping of people. As a result, the comparison between the overall agricultural and non-agricultural populations cannot yield an accurate picture of the differences in occupational mobility between RMW and URW, the agricultural and non-agricultural populations working and residing in the urban areas.

The current scholarship on inequality of occupational attainment between RMW and URW is largely dominated by studies that focus on occupational segregation in the urban labor market. The area of equality of occupational mobility between RMW and URW, which may further reveal RMW's risks of employment and economic insecurity and disadvantage of economic life chances enhancement, is still under researched. In particular, there is a lack of scholarship comparing RMW's and URW's occupational mobility in a fixed period of time following a comparative social mobility analysis approach. This brief research report aims to fill this gap by investigating the differences in occupational mobilities between RMW and URW in China's urban labor market. Drawing on data from the China Family Panel Studies (CFPS), this study uses three heatmaps to visualize the relative differences in occupational mobility between RMW and URW within the periods 2010–2014, 2014–2016, and 2010–2016 based on the outflow table comparison method.

## Methods

### Data Source

This study uses data from the China Family Panel Studies (CFPS) (Institute of Social Science Survey, Peking University, 2015), a large-scale, nationally representative and longitudinal survey of Chinese communities, families, and individuals, containing a wide range of variables (Xie and Hu, 2014). As a panel study, CFPS has documented individuals' information of residence, employment, occupation and *hukou* type overtime, which are essential for the study of comparative occupational mobility. Currently, there are five waves of data available (2010, 2012, 2014, 2016, and 2018). Among them, adult datasets at wave 2010, 2014, and 2016 contain good quality information of individuals' occupations. Therefore, data from CFPS2010adult, CFPS2014adult, and CFPS2016adult were used to compare occupational mobilities within the period 2010–2014, 2014–2016, and 2010–2016. However, this study retains a limitation of only focusing on a specific period, due to the availability of data.

### Sample

In accordance with the target population (i.e., employed workers in urban areas at the earlier wave), this study selected the urban-living and non-agricultural job cases at the earlier wave of each unit of analysis as the valid sample for the study. Table 1 summarizes the process of valid sample selection. In the beginning, 5,457 eligible cases were selected for the 2010–2014 and 2010–2016 periods and 6,226 for the 2014–2016 period. The latter two steps are aimed at dealing with the missingness. As shown in Table 1, the issue of item-missing is not serious, but the wave-missing rates are not very low. This study conducted a complete case analysis after listwise deletion. Although the sample sizes are still large enough for the statistical analysis, this study retains a potential limitation of sample bias, as it is usually naive to assume that the missing would be completely at random.

**Table 1:** The process of valid sample selection.

		**2010–2014**	**2014–2016**	**2010–2016**
Step one: Eligible cases selection	1. Total nationally representative sample from wave a (within the “a to b” period)	33, 600	34, 731	33, 600
	2. Eligible sample selection: urban-living and employed as non-agricultural worker	5, 457	6, 226	5, 457
Step two: Missing value of *hukou* at wave a		5, 453 (4 items missing, 0.07%)	6, 179 (47 items missing, 0.75%)	5, 453 (4 items missing, 0.07%)
Step three: Attrition, and item missing at wave *b*	1. Remain at wave b	3, 695 (1, 250 dropouts, 22.9%; 508 individual questionnaires not given, 9.32%)	4, 999 (659 dropouts, 10.67%; 521 individual questionnaires not given, 8.43%)	3, 458 (1, 457 dropouts, 26.72%; 538 individual questionnaires not given, 9.87%)
	2. Employment status at wave b	3, 159 (57 items missing, 1.54%; 479 economically non-active, 12.96%)	4, 328 (149 items missing, 2.89%; 522 economically non-active, 10.44%)	2, 791 (102 items missing, 2.95%; 565 economically non-active, 16.34%)
	3. Among the employed, valid information about occupation at wave b	3, 035 (originally: 347 items missing, 11.13%; After imputing 266 values from wave 2016: 81 items missing, 2.67%)	4, 213 (46 items missing, 0.1%)	2, 733 (26 items missing, 0.94%)
	So, economically active (valid information about occupation at wave b+ unemployed)	3,078	4,282	2,765
	Valid sample	3,078	4,282	2,764 (dropped one more case without information of longitudinal weight)

### Variables

To indicate occupational class, this study used a variable measuring individuals' occupation category at each wave based on the Erikson–Goldthorpe–Portocarero (EGP) schema (Erikson et al., 1979). The EGP scheme is considered as a useful tool to measure occupational class, especially for the purpose of comparing social mobility in terms of economic life (Atkinson, 2015, p. 50–53; Goldthorpe, 2016). Although the EGP scheme was originally used in the context of western industrial societies, Zou (2015) study shows that the scheme has a good constructive validity in measuring individuals' economic life chances in China's urban labor market despite the different economic and political institutions. In practice, leading empirical studies in comparative social mobility in China (e.g., Wu and Treiman, 2007; Zhou and Xie, 2019) also preferred to adopt this scheme. After some adjustments, this study created a nine-category class scheme^1^ that also includes the unemployed but excludes those who had no intention to seek a job in the labor market at the later wave of the analysis.

The datasets contain a variable measuring individuals' *hukou* type (agricultural or non-agricultural). As all the selected cases were living in urban areas when being survived, those who still had an “agricultural” *hukou* type were classified as RMW, whilst the “non-agricultural” *hukou* holders were URW.

### Analytical Methods

**Figure 1:**
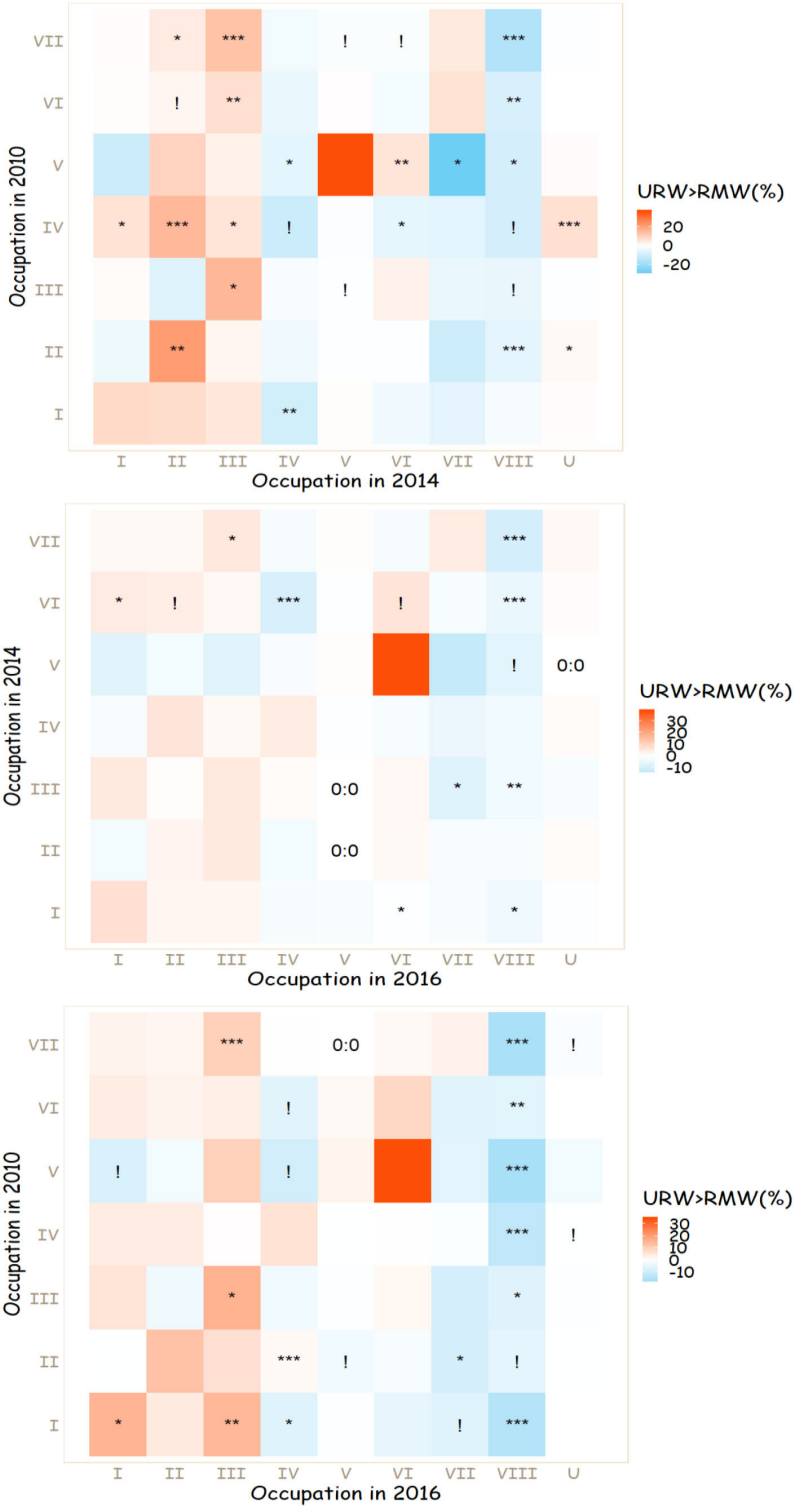
Heatmaps on comparative occupational mobilities between RMW and URW (Redness stands for URW having a higher cell percentage and blueness stands for RMW having a higher cell percentage) with Chi-squared test result (***p < 0.001, **p < 0.01, *p < 0.05, ! *p* < 0.1, 0:0 no case in both RMW and URW groups). I, Higher managerial and professional; II, Lower managerial and professional; III, Routine non-manual; IV, Self-employed; V, Manual supervisor; VI, Skilled manual; VII, Semi-unskilled manual; VIII, Agricultural; U, Unemployed; Source: CFPS adult 2010, 2014, and 2016.

The study is based on comparing RMW's and URW's outflow distributions of occupational mobility. Firstly, this study creates outflow tables of occupational mobilities for RMW and URW samples, respectively. An outflow table is simply a two-way table (the original occupation in the row, the destination occupation in the column) that shows the row percentages of entering different occupational destinations for those who originate from the same occupational class. But because the main purpose is to compare RMW's and URW's occupational mobilities, the comparison actually goes three ways. After having RMW's and URW's outflow tables, their cell percentages were compared by making subtractions for each cell. Based on the substation results, three heatmaps were created to visualize the RMW-URW difference of each cell's percentage, which is shown in Figure 1.

Additionally, Chi-squared test results are also attached (see Figure 1) to indicate whether the conditional RMW-URW difference also seems reliable in the population. Every unit of a test was based on a two-by-two table grouped by *hukou* type (row) and a binary outcome of entering a certain occupational class at the later wave (column), for those who were from the same original occupation at the previous wave. Most analyses and plotting were done using Stata and R package *ggplot2*.

## Results

The results are presented in three heatmaps (Figure 1). From top to bottom, the heatmaps show the comparisons within the period of 2010–2014, 2014–2016, and 2010–2016. For each cell in each heatmap, red represents the percentage in the URW group was large, whilst blue represents that RMW's percentage was relatively high. Furthermore, asterisks were used to show the Chi-squared test results on the RMW-URW differences.

For all of the heatmaps, the left side of the diagonal line shows upward mobility, and the right side of the diagonal line shows downward mobility. In each of the graphs, there is more red shading than blue on the left side of the diagonal lines, meaning that URW had a higher chance of success in upward mobility. On the other hand, on the right side of the diagonal lines, which represents downward mobility, there is show more blue shading than red shading during all periods indicating that RMW were more likely to experience downward mobility than URW.

At first glance, for all three heatmaps, the first three columns are dominated by red shades, meaning that regardless of their original occupations, URW were more likely to take over the “white-collar professions” (i.e., I, II, and III) than RMW. In contrast, within all the periods, the “blue-dominance,” located on the right-hand side of the heatmaps, especially around the columns of semi-unskilled manual and agricultural, suggests a greater tendency for RMW to move to or stay at the lower level occupations, regardless of their original occupations. In addition, with regards to becoming agricultural workers, the “VIII” column shows a consistent blueness over all the observed periods with many cells showing a statistically significant difference, indicating that RMW from all of the occupation groups had a much stronger tendency to become agricultural workers (presumably in rural areas) compared to those who held a non-agricultural *hukou* status (i.e., URW). However, the last column does not show any clear pattern of RMW-URW disparity in becoming unemployed.

Longitudinally, the “red-dominance” on the first three columns and the dominance of blue shades on the right of the graphs are more apparent within the overall context of the 6 years (2010–2016) whilst being less salient within only 2 years (2014–2016). This suggests that the RMW-URW gap in mobility was more noticeable in a longer period.

Among those who were from managerial and professional occupation backgrounds, URW were more likely to secure their previous positions than their rural migrant counterparts. When experiencing downward mobility, URW with higher managerial and professional backgrounds had a higher chance in securing a white-collar occupation (i.e., II and III). In contrast, even if they were starting from a managerial or professional position, rural migrants had a relatively high risk of downgrading to a semi-unskilled manual labor occupation, especially between 2010 and 2016 when the difference was estimated to be statistically significant. Within the periods 2010–2014 and 2010–2016, higher managerial and professional RMW were much more likely to become self-employed workers.

Within those intermediate categories (i.e., III, IV, and V), the category of manual supervisor is often thought to have a close relationship with the manual labor occupations. Regarding the mobility of the manual supervisors, it seems that, in a relative sense, the urban resident manual supervisors exhibited a strong likelihood to stay as manual supervisors (as noted in the red shades in 2010–2014 and 2010–2016) or at least to be skilled manual workers (red shades across all the periods and a significant difference within the first 4 years) but a small chance of becoming semi-unskilled manual workers (as noted in the blue shades across all the periods and a significant difference within 2010 to 2014). However, there are some signs which suggest that more rural migrant manual supervisors are taking over the higher managerial and professional positions, indicated by the blue shading in all the periods and a significant difference (but only at 90% level) within 6 years. Whilst the pattern in the 2014–2016 period is less clear than in the other observed time periods, the URW, who started as self-employed individuals, were more likely to take over managerial and professional positions than their RMW counterparts and the difference is estimated to be significant in the first 4 years. Regarding routine non-manual background individuals' mobility, URW did not show a clear advantage in further upward mobility to the managerial and professional class, yet they still did a better job in securing a routine non-manual position (especially in the 2010–2014 and 2010–2016 periods) and had a smaller risk of downgrading to a semi-unskilled manual or agricultural labor occupation.

There is no clear pattern of the RMW-URW differences in manual background workers' staying in manual labor occupations, but there seems to be some consistency in the difference in their upward movement trends. For those whose backgrounds were semi-unskilled manual labor occupations, URW consistently had a significantly higher likelihood of becoming routine non-manual workers across all three periods. Moreover, urban resident skilled manual workers tended to take a higher proportion of routine non-manual and lower managerial and professional jobs in the first 4 years (2010–2014), and they were more likely to enter the managerial and professional class in the following 2 years (2014–2016). By comparison, rural migrant manual workers' upward mobility seems to be marked by a trend in self-employment. This echoes some previous research on rural migrant manual workers' upward mobility trends which also found that they turned to self-employment as a common approach in achieving upward mobility (Li, 2004, p. 108–150; Zimmermann et al., 2011; Cui et al., 2013).

## Discussion

Whilst previous literature fails to provide empirical evidence of equality between RMW's and URW's occupational mobilities, this brief research report makes a contribution to the field by updating the findings on the differences in occupational mobility between RMW and URW in urban China. Clearly, the result suggests that in terms of occupational mobility, RMW were at a notable disadvantage compared to URW. Regardless of their original occupation, URW were at an advantage when it came to obtaining white-collar positions, especially managerial or professional positions. In comparison, RMW were more likely to stay in or move to manual occupations. In addition, RMW were also much more likely to become agricultural workers once again. Also, the study did not find a difference between RMW and URW in becoming unemployed.

The most striking finding of this study is the clear “white-collar/blue-collar” divide between RMW and URW. This updated finding enriches the understanding of the nature of the occupational inequality between RMW and URW in urban China. A large body of literature has already evidenced the occupational segregation over manual/non-manual barrier between RMW and URW (e.g., Meng and Zhang, 2001; Li, 2004, p. 42; Zhang and Wu, 2017). The predominant explanation given for this is that the newly arrived rural migrants usually take over the low-skilled manual labor jobs in the manufacturing and construction sectors in cities, whereas those who have resided in cities for long periods of time generally do not take these up, even if these jobs are (e.g., Li, 2004; Chan, 2010). However, the findings of disparity in mobility in this study supports an additional mechanism at work that leads to a “white-collar/blue-collar” divide. According to the findings of this study, even though some RMW cases started from a managerial, professional, or routine labor non-manual position, the risk of downgrading to manual workers (especially to semi-unskilled manual workers) was relatively high. This suggests that the occupational segregation is at least partly due to the RMW-URW difference in employment security. Li (2004, p. 156) argues that RMW's employment is more akin to a “completely free market principle” when selling and buying their labor power. This “free market” then leads to RMW's increased risk of job insecurity (Ibid.). On the other hand, there were some URW who first worked in low-skilled manual labor jobs and then they managed to shift to non-manual role more quickly than RMW.

Moreover, the findings in this study provide new quantitative evidence demonstrating that rural migrant manual workers are more likely to become self-employed. For manual workers' upwardly mobile pathways, whilst URW had a higher chance to become white-collar workers, RMW were more likely to become self-employed. This echoes previous findings (Li, 2004, p. 108–150; Zimmermann et al., 2011; Cui et al., 2013) which indicated that becoming self-employed is usually the most common approach in achieving an improved socio-economic status for rural migrants whose first jobs as employed manual workers in the urban labor market. On the one hand, becoming self-employed is certainly helping rural migrant manual workers avoid a low-pay job in cities (Cui et al., 2013). On the other hand, the lack of educational resources in rural areas and the *hukou*-based system of discrimination in the recruitment process to some privileged jobs hinders rural migrants from being employable in the labor market of skilled and professional occupations (Li, 2004, p. 168; Cai and Chan, 2009). Consequently, self-employment becomes a desirable and practically achievable career progression option as compared to other upwardly mobile pathways for RMW.

Interestingly, while the results show the RMW-URW inequality of occupational mobility, there is no signs of RMW's higher risk of unemployment. These findings echo previous research findings on RMW's low unemployment rate (Li, 2008). However, a low unemployment rate does not mean a lack of job insecurity, since RMW change jobs frequently in order to keep themselves being employed (Li, 1999, 2004, 2010; Zhang, 2011; Fu and Tang, 2016). On the demand side, with no entitlement to welfare benefits in cities, RMW cannot afford to be unemployed for long (Chan, 1996, 1999; Li, 2008). On the supply side, there are plenty of short-term unskilled jobs available for RMW in the urban labor market, and most of the jobs do not even offer a signed labor contract to the workers (Li, 2008).

In addition, the disparity could also be related to the difference in *hukou* conversion for those who were originally from rural areas, as *hukou* conversion and socio-economic life-chances improvement are highly correlated. Socio-economically better-off occupations, especially the jobs in the state sector, usually allow rural background workers to change their *hukou* type into local non-agricultural status easily (Chan, 1996). In this study, RMW is defined as those who had migrated to work in urban areas but still hold an agricultural *hukou* type. Within the managerial and professional category, there might be some internal differences. For example, the managerial or professional jobs that RMW have might be less advantageous than the jobs of those who were originally from rural areas but the RMW managed to gain a non-agricultural *hukou* status and become a *de jure* urban resident. This might partly explain agricultural managerial and professional workers' relatively high chance of downward mobility. As such, the findings suggest future research into the *hukou* effect on occupational mobility and to pay more attention to the role of *hukou* conversion in employment mobility.

Despite its contribution to the existing literature, this study contains some limitations. Firstly, the main objective of this brief research report is to briefly update an empirical investigation about inequality of occupational mobility between RMW and URW in urban China in order to fill the gap in the literature, whereas the gap of in-depth theoretical discussion needs to be filled by future research. Secondly, being subject to the availability of data, this study only provides evidence of the comparison of mobility between RMW and URW on a specific period, from 2010 to 2016. Thirdly, as a brief research report, whilst this paper has provided a robust comparative analysis of the occupational mobility differences between RMW and URW, there is a lack of further step explanatory analysis to explore the factors (e.g., sample characteristics) contributing to the observed inequality of mobility. As such, future research could attempt to decompose the factors explaining the RMW-URW differences in occupational mobilities.

## Data Availability Statement

Publicly available datasets were analyzed in this study. This data can be found at: https://opendata.pku.edu.cn/dataverse/CFPS?language=en.

## Author Contributions

The author confirms being the sole contributor of this work and has approved it for publication.

## Conflict of Interest

The author declares that the research was conducted in the absence of any commercial or financial relationships that could be construed as a potential conflict of interest.

## Footnotes

^1^Categories: 1, Higher managerial, professional and administrative; 2, Lower managerial, professional and administrative; 3, Routine non-manual; 4, Self-employment; 5, Manual supervisor; 6, Skilled manual; 7, Semi-unskilled manual; 8, Agricultural workers; 9, unemployed.
